# Carbon-Based Functional Nanomaterials as Tools for Controlling the Kinetics of Tribochemical Reactions

**DOI:** 10.3390/ma17040785

**Published:** 2024-02-06

**Authors:** Dariusz Ozimina, Andrzej Kulczycki, Dawid Janas, Tomasz Desaniuk, Maciej Deliś

**Affiliations:** 1 Faculty of Mechatronics and Mechanical Engineering, Kielce University of Technology, Tysiąclecia P.P. 7, 25-314 Kielce, Poland; 2 Air Force Institute of Technology, Ostroroga 35a, 01-163 Warszawa, Poland; 3 Department of Chemistry, Silesian University of Technology, B. Krzywoustego 4, 44-100 Gliwice, Poland; 4 GE Aerospace Poland Ltd., Krakowska 110/114, 02-256 Warsaw, Poland

**Keywords:** graphene, CNTs, Au-decorated CNT, fullerenes, triboreactions, lubricity additives

## Abstract

The aim of this article is to experimentally determine the role of the environment, consisting of a base oil (PAO), carbon nanomaterials, and optional other additives, as well as the kind of metal in contact with the lubrication film, in the stimulation of zinc dialkyldithiophosphate (ZDDP) additives’ effectiveness during protective film formation. This paper focuses on the role of carbon nanostructures in energy transportation and conversion during tribological processes. An antistatic additive (ASA) (not used in lubricating oils) for jet fuels was added to disturb the process of energy conduction (electric charges) through the lubricant film and thus determine how this disturbance affects the kinetics of the ZDDP triboreaction and, consequently, the linear wear. To achieve this research goal, two types of tribological testing devices were used: an Anton Paar tribometer (TRB) and a triboelectric tribometer (TET). The novelty of the present research is in the use of the method for disturbing the flow of charge/energy through the lubricant film with an antistatic additive for jet fuels, ASA, to influence the impact of this energy on the antiwear properties of ZDDP. The following conclusions were drawn: (1) carbon-based nanostructures, i.e., CNTs, AuCNTs, graphene, and fullerenes, are able to change the rate of chemical reactions of ZDDP during tribological processes; (2) CNTs have the ability to catalyze tribochemical reactions of ZDDP, while graphene and fullerenes are not able to perform this effectively; (3) AuCNT takes the role of an inhibitor during ZDDP’s triboreaction; and (4) by discharging electric charge/energy, ASA, in cooperation with CNT and AuCNT significantly reduces the rate of the ZDDP reaction.

## 1. Introduction

Efficient friction and wear reduction are possible when tribochemical processes are properly controlled; this requires knowledge of the mechanisms behind these processes. In the 1990s, Yao and Dong observed tribocatalytic interactions of antiwear additives in complex tribological systems [1,2].

The possibility of applying knowledge of the mechanisms of heterogeneous catalysis to tribochemistry seems to be very attractive. This application will be possible if tribochemical processes are treated as catalytic reactions.

The above concept is consistent with the proposals for the mechanisms of tribocatalytic reactions described in the authors’ earlier publications. Below, the concepts of the mechanisms of tribocatalysis, compared with the concepts formulated on the basis of reactivity model α_i_, are shown.

The authors of [3] describe the current views on the mechanism of protective film formation by ZDDP lubricating additives. They wrote that ZDDP additives are not stable compounds and easily decompose during friction. ZDDP additives’ tendency toward decomposition can be explained by chemical principles called “hard” and “soft” acids and bases. The bases are reactive nucleophiles, and the acids are electrophiles. They can be treated as “hard” and “soft” according to their charge and polarizability. The “hard” and “soft” acid and base theory states that a “hard acid” tends to react with a “hard base” and a “soft acid” tends to react with a “soft base.” This theory seems to be useful for explaining the influence of tribochemical reactions’ environment on the kinetics of ZDDP decomposition and protective film formation. It can be concluded that the “hard” and “soft” acid and base theory can be adopted to explain how the reaction environment influences energy transfer inside the tribological system and its impact on the kinetics of triboreactions.

The authors of [4] formulated the following hypotheses:

- Tribocatalysts convert the mechanical energy in the stream of electrons and/or photons, which provides additional energy to the molecules of reacting substances (lubricating additives).
- A chemical reaction can be started when the molecules of a reacting substance are supplied with energy equal to the activation energy E_a_ and energy is provided in a sufficiently large stream.

They used reactivity model α_i_ to describe the tribological process [5,6]. This model is expressed by the following equations:

α_i_ = (L − L_o_) C (1)

(2) $$C=\frac{1}{A_{k}K}.$$

These hypotheses were verified by the research described in [6], which was focused on analyzing the behavior of lubricants containing zinc dialkyldithiophosphate (ZDDP) and ordered molecular structures such as carbon nanotubes (CNTs), fullerenes (C60), and an antistatic additive (ASA). These carbon nanomaterials, as well as ASA, an additive that increases the electrical conductivity of the lubricant, have been used to modify ZDDP’s environment during triboreactions. It was found that the effectiveness of the lubricating additive (ZDDP) depended on the presence of ordered molecular structures, which were able to conduct energy from the surface of the specimen to ZDDP reaction zones inside the boundary lubricating film. CNTs and ASA were reported to conduct energy effectively. Fullerenes (C60), on the other hand, were not suitable energy conductors in the lubrication process.

The aim of this study was to experimentally determine the role of the environment, consisting of base oil (PAO), carbon nanomaterials, and optional other additives, as well as the kind of metal in contact with the lubrication film, in the stimulation of ZDDP additives’ effectiveness during protective film formation. It was assumed that the lubricity additives’ effectiveness in protective film formation depends on the triboreaction rate. The results of this study can be useful for better selection of the chemical composition of lubricating oils and of construction materials for device elements that participate in tribological processes.

During friction, the molecules of the lubricating additive are subjected to the action of solids, as well as the components present in the protective layer created by the lubricant. A large body of literature data suggests that lubricity additives undergo tribocatalytic reactions [7,8,9,10,11,12,13,14,15,16]. In this case, the triboreaction rate depends not only on the heat generated during friction but also on the tribocatalytic effect. The previous research described, including in [17], indicates the three stages of the tribocatalytic process:

- Converting the mechanical energy introduced into the tribological system to the energy of hot electrons emitted by the solid surface to the lubricant layer;
- Transporting this energy inside the lubrication film and converting it into a form suitable for the chemical reaction’s initiation;
- Supporting the molecules of reactants (lubricating additives) with this energy and the chemical reaction’s initiation.

The first stage depends on the conditions of the tribological process and the material of the solid elements of the tribological system. The second stage depends on the components of the lubricant, including the environment of the reactant molecules. The third stage depends on the properties of these components of the lubricant, which are responsible for the additive molecules’ support with energy. Some research indicates that the acid–base theory can be used to explain CNTs’ influence on the triboreaction rate [18].

Taking into account the “hard” and “soft” acid and base theory, the role of metals in carbon nanotubes’ decoration in tribocatalytic reaction rate stimulation was examined. It was assumed that carbon nanostructures can take on an important role in energy transportation from the space near the surface of the solid to the additive molecules inside the protective film. The metals used in CNT decoration can interact with CNTs’ carbon structure, making it easier or more difficult to absorb energy and then transport it to the reaction zone.

This paper focuses on the role of carbon nanostructures in energy transportation and conversion during the tribological process. The ASA (not used in lubricating oils) for jet fuels was added to disturb the process of energy conduction (electric charges) through the lubricant film and thus determine how this disturbance affects the kinetics of the ZDDP triboreaction and, consequently, the linear wear.

## 2. Methods

To achieve the research goal, i.e., experimental determination of the role of the environment, consisting of base oil (PAO), carbon nanomaterials, and optional other additives, as well as the kind of metal in contact with the lubrication film, in the stimulation of ZDDP additives’ effectiveness during protective film formation, two types of tribological tests were used.

One is a test on the TRB apparatus, in which the influence of the chemical composition of the lubricating oil on linear wear was precisely determined. After each test, the cooperating elements, i.e., the ball and the disc, were tested for the content of Zn, P, and S, which can only come from the ZDDP. The concentration relationships of these elements, other than those in the ZDDP molecule, indicate that on the surface of wear scars on the ball and disc, there were products of the chemical reaction of the additive.

The second test was conducted on the triboelectric test (TET) tribometer. This apparatus allows for research on the influence of tribochemical processes taking place in the lubricant film in terms of the flow of electrical charges and the voltage generated. Using pins made of different metals, it is possible to determine the functional relationship between voltage and the normal potential of the metal for each tested lubricating oil and then observe how a change in the composition of the lubricating oil affects the function. In the present study, this test was used to investigate how the addition of ASA affects this function.

**Figure 1:**
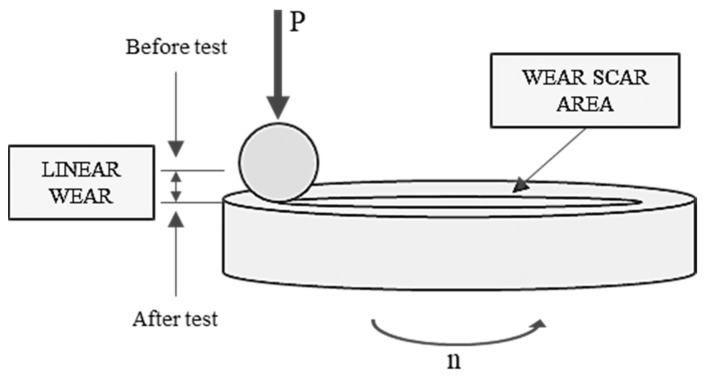
Tribological measurements.

The tribological tests were performed utilizing a TRB^3^ tribotester operating in a ball-on-disc configuration. As shown in Figure 1, the tribological tests involved measuring the coefficient of friction, linear wear, and the wear scar surface area.

The values measured with two friction sensors mounted on the TRB^3^ tribometer were saved automatically. The experiments were carried out under the following conditions:

- Load (P): 5, 10, 30, 50 N;
- Sliding speed (v): 0.1 m/s;
- Sliding distance (s): 1000 m;
- Humidity: 25 ± 5% RH;
- Ambient temperature (T_0_): 25 ± 4 °C;
- Elements in contact: 100Cr6 steel balls and HS6-5-2C steel discs, with the latter uncoated or coated with a-C:H.

In this study, the test result was the linear wear (LW). Moreover, the content of Zn, P and S, expressed in wt.% on the surface of friction elements, was analyzed using the EDS method. The analyzed concentration of Zn, P, and S is the average value from measurements in 10 fields in the wear trace. The content of these elements was treated as the measure of ZDDP’s triboreaction products, deposited on the surface of the ball and disc.

**Figure 2:**
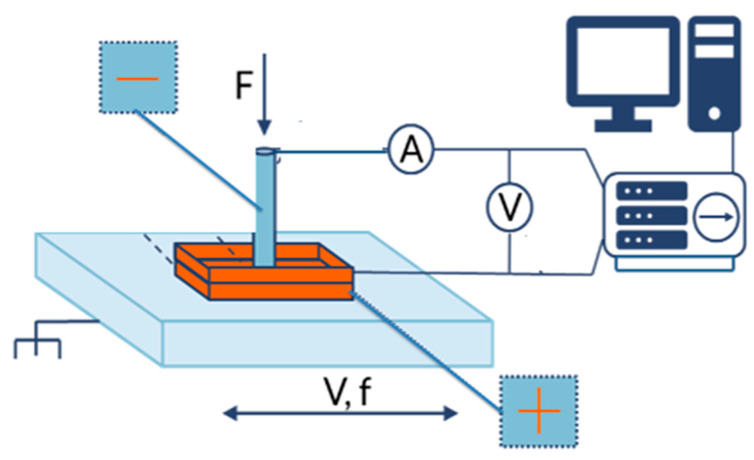
Diagram of the experimental setup for TET.

The second test during experimental studies was the TET. The experimental setup used to measure the electricity generated by friction consisted of a drive system with a head for holding the upper specimen and a sliding table with the lower specimen holder. The experiments involved moving the lower specimen (plate) reciprocally so that it was in sliding contact with the stationary upper specimen (pin). The test setup with the pin-on-plate configuration is shown in Figure 2.

The testing device was equipped with dedicated software, which made it possible to control the stroke length, frequency of oscillation, acceleration, and sliding speed. It operates continuously without switching off or offloading.

The TET test conditions were as follows:

- Frequency of oscillation: 0.5 Hz;
- Load: 5 N;
- Stroke length: 40 mm;
- Maximum sliding speed: 0.28 m/s;
- Acceleration: 2 m/s^2^;
- Braking speed: 2 m/s^2^.

Voltage (U) was registered as the measure of energy flow during the tribological process. This voltage was used as the measure of lubricant additives’ reactivity due to the influence of the chemical reaction of ZDDP on the energy flow through the lubricant film.

## 3. Materials

The lubricating additive ZDDP was used as the reagent undergoing tribocatalytic reactions. This kind of additive is widely used to improve lubricity (and especially the antiwear properties) of engines, gears, and hydraulic oils. This additive was dissolved in PAO8 base oil. Various carbon nanomaterials of different structures (CNTs, C60, graphene), as well as AuCNTs [19], were added to the lubricating oil consisting of PAO8 and ZDDP to modify the environment of the reagent. It was interesting to observe how Au changed the tribochemical properties of CNTs. In prior studies, AuCNTs were synthesized in a laboratory at the Silesian University of Technology by Prof. D. Janas. Additionally, to verify the hypothesis of the influence of energy distribution in the reaction environment on the kinetics of the reaction, the ASA was added [20]. The lubricants used during the tribological tests are described in Table 1.

The properties of the materials are as follows:

- PAO8—polyalphaolefin 8; a commercial product with a specific density of 833 kg/m^3^ and a kinematic viscosity at 100 °C of 7.8 mm^2^/s;
- ZDDP—zinc dialkyldithiophosphate with primary alkyl groups; a commercial product with a density of 1160 kg/m^3^ and a kinematic viscosity at 40 °C of 150 mm^2^/s, containing 9.0% by weight of Zn, 8.5% by weight of P, and 16.5% by weight of S;
- ASA—the commercial antistatic additive for jet fuels, consisting of a C/H/O/S polymer + polyamine + R-S0_3_H stabilizer;
- CNT—carbon nanotubes; a high-purity (>85%) commercial product made by CARBON4nano; industrial grade single-walled, unmodified carbon nanotubes with an average diameter of 1.6 nm and a length of 5 µm; impurities with organic substituents containing O, Al, and Fe bonded with the carbon atoms;
- AuCNT—carbon nanotubes decorated by Au;
- C60—fullerenes; a high-purity (>99.9%) commercial product with a density of 1.65 g/cm^3^;
- Graphene—obtained from AGP Advanced Graphene Products; carbon content 99.8%; flake size (DLS method): average 500 nm, >90% with a diameter less than 800 nm; number of layers <10.

## 4. Results

### 4.1. TRB Test Results

**Figure 3:**
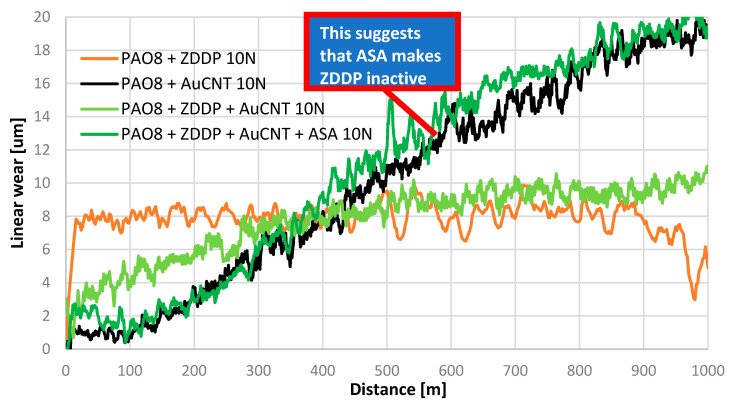
The results of TRB tests: linear wear vs. friction distance; load 10 N.

The first part of the research was focused on the interaction between ZDDP and AuCNT. The linear wear was used as the criterion for judging ZDDP’s activity as a lubricity additive. The results of the TRB tests under a load of 10 N are shown in Figure 3.

The above results indicate that AuCNT added to PAO8 without ZDDP significantly decreased linear wear during the first stage of the test, but the linear wear increased during the test, reaching a value of 20 μm by the end.

The linear wear obtained for PAO + ZDDP was highest during the beginning stage of the TRB test and was stable during the test, achieving a value of 8 μm. Our experience, based on previous studies on TRB, led us to conclude that the mechanism of lubricating film formation during the beginning stage of the test was different than that during the final stage. During the beginning stage, triboreactions of lubricity additives increased linear wear. On the contrary, during the final stage, triboreactions decreased linear wear.

During the beginning stage, the behavior of PAO + ZDDP + AuCNT and PAO + AuCNT was similar. After a distance of 400 m, the linear wear reached a value of 9 μm and then was stable.

Interesting results were obtained for PAO + ZDDP + AuCNT + ASA. The value of linear wear for this lubricant changed when, for example, PAO + AuCNT was obtained. This suggest that ASA in interaction with AuCNT makes ZDDP inactive.

The above results can be compared with the experimental data presented in Table 2.

These data indicate the important role of Au, which significantly changed the catalytic effect of carbon nanotubes. Compared to PAO + ZDDP, the CNT rapidly decreased linear wear, while AuCNT increased it slightly.

**Figure 4:**
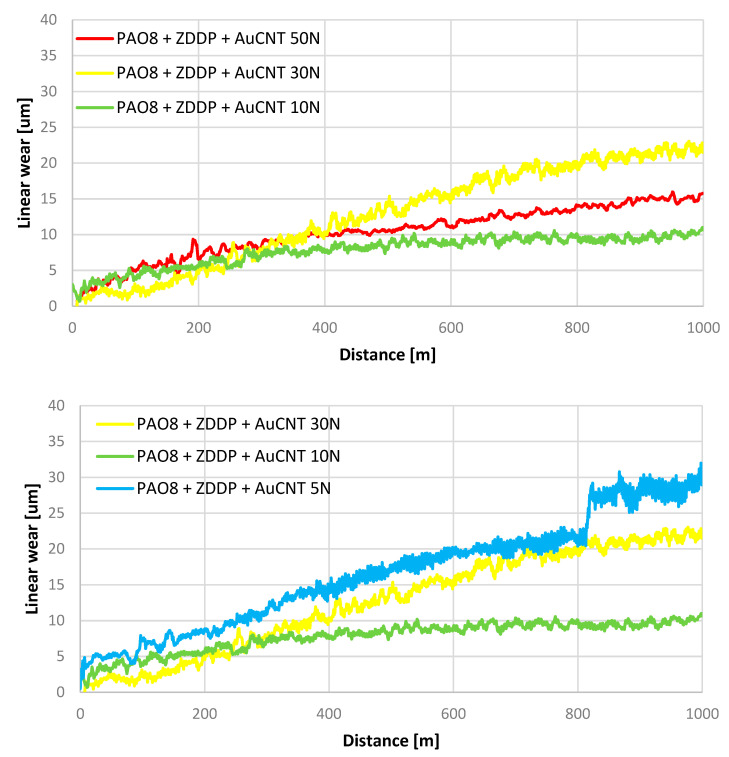

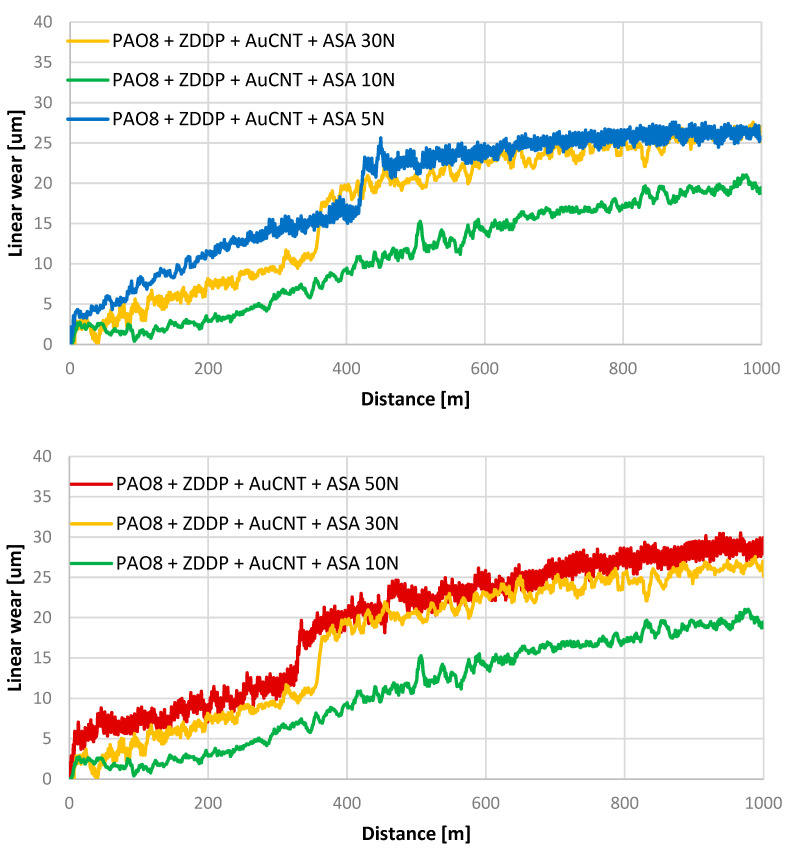
Linear wear vs. applied load.

Figure 4 shows the influence of applied load on linear wear for two lubricants: PAO + ZDDP + AuCNT and PAO8 + ZDDP + AuCNT + ASA.

### 4.2. Investigation of the Effect of a Solid Material on the ZDDP Kinetics of Triboreaction—TET Test Results

**Figure 5:**
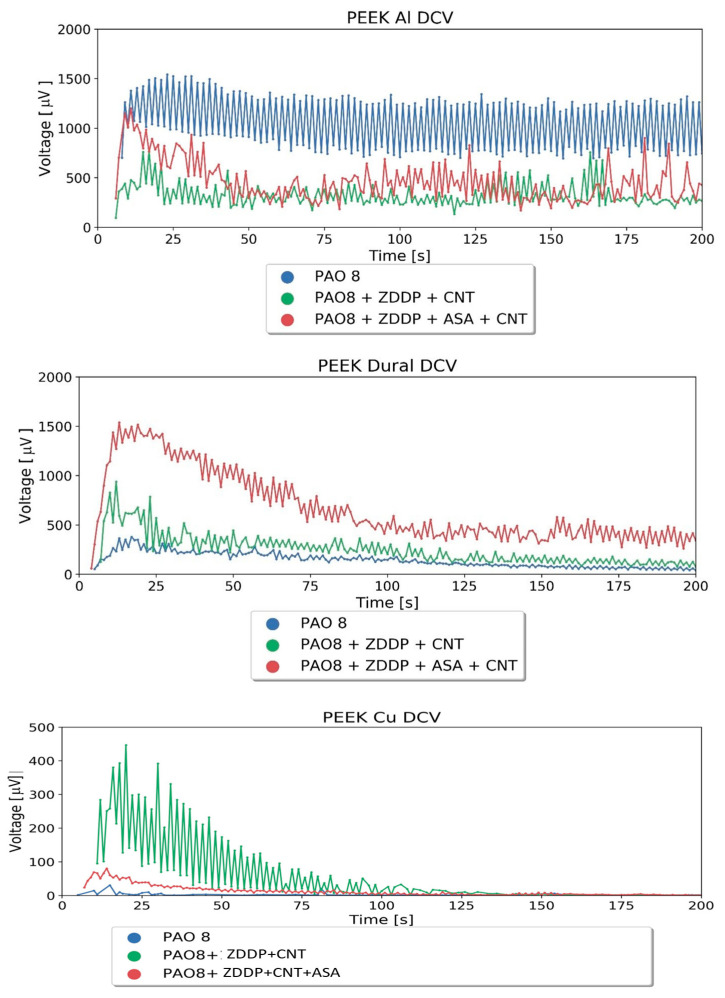
Voltage measurement results during TET tests.

The next part of the investigation was focused on triboelectric effects as a measure of energy distribution in the lubricating film during friction. The influence of solid materials on the amount of energy generated during friction was the core topic of the TET tests. Dural aluminum and copper were used as materials for the pins in the TET stand. PEEK was used as the material for co-samples. The co-samples were cuboid, with dimensions of 70 × 30 × 10 mm, and placed 2 mm deep in the tested oil. The material of the co-samples (polymer) was chemically inactive towards PAO8 and the ZDDP additive. Three kinds of lubricants were used: PAO, PAO + ZDDP + CNT, and PAO + ZDDP + CNT + ASA. Figure 5 shows the results of the voltage measurements during the TET tests.

**Figure 6:**
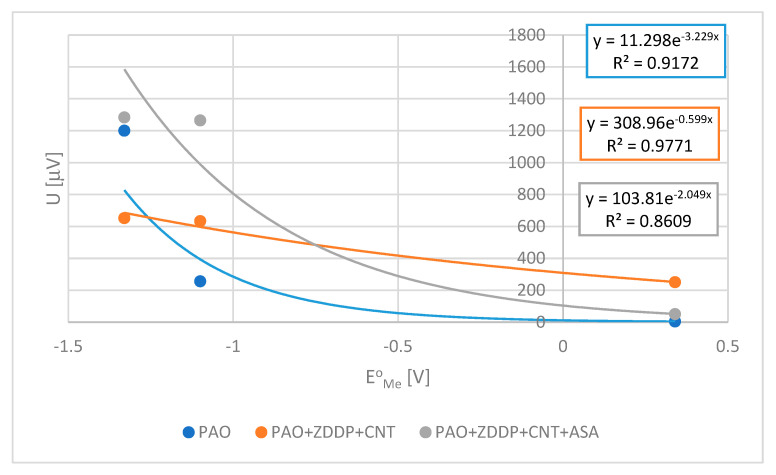
The relationships between voltage U during TET triboelectric tests and the values of normal potential of the pin material E°_Me_ used.

Table 3 shows the voltage measured after 25 s. Figure 6 presents the empirical relationships between voltage U and normal potential E^o^_Me_ determined for lubricants consisting of PAO8 and containing ZDDP + CNT and ZDDP + CNT + ASA.

The experimentally obtained trend lines can be described by the following exponential relationships.

The experimentally determined function, shown in Table 4, indicates the significant role of CNT in charge transportation from the lubricant to the solid material and ASA additive, which significantly limits this effect.

## 5. Discussion

The above results of TRB tests were assessed using the reactivity model α_i_.

### 5.1. The Load Influence on Linear Wear

In relation to linear wear, it was assumed that work done on tribological system L can be expressed as follows:

(3) L = WL_wo_ P

where W is the linear wear (μm), P is the applied load (N), and L_wo_ is the work that has been done to obtain the unit wear under unit load (J/(μm N)). In this case,

α_i_ = (W L_wo_ P − L_o_) C

After transforming the above formula, we obtain

(4) $$\frac{\frac{\alpha_{i}}{C}+L_{o}}{L_{wo}P}=W$$

Differentiation of Equation (4) gives

$$\frac{\frac{\alpha_{i}}{C}+L_{0}}{L_{wo}}\frac{1}{P}dP=dW$$

After integration, we then obtain

(5) $$\frac{\frac{\alpha_{i}}{C}+L_{o}}{L_{wo}}\left(lnP_{1}-lnP_{2}\right)=W_{1}-W_{2}$$

**Figure 7:**
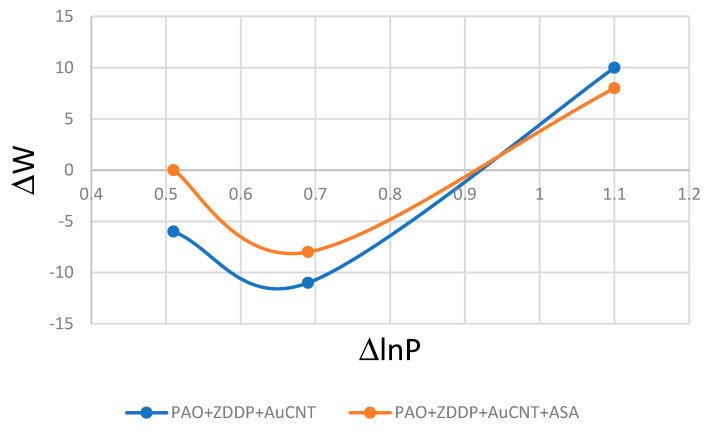
The relationship between ΔW and ΔlnP obtained during TRB tests.

Based on the data shown in Figure 3, ΔW and ΔlnP were determined for lubricants PAO8 + ZDDP + AuCNT and PAO8 + ZDDP + AuCNT + ASA. The results are presented in Figure 7.

### 5.2. The Influence of Carbon Nanostructures on the Kinetics of ZDDP’s Triboreaction during TRB Tests 

It can be assumed that, during the tribological process, ZDDP undergoes the following reaction:

ZDDP → D = ∑D*_Zn_* + ∑D*_P_* + ∑D*_S_*

where D is the deposit on the surface of the ball and disc, ∑D*_Zn_* is the deposit on the ball and disc consisting of *Zn*, ∑D*_P_* is the deposit on the ball and disc consisting of *P*, and ∑D*_S_* is the deposit on the ball and disc consisting of *S.* This reaction, named in [3,21] as the decomposition of ZDDP, leads to protective layer formation. The structure of this layer consists of the part in contact with Zn and the part in contact with S and P. The whole of this layer is responsible for linear wear. As shown in Figure 3 and Figure 4, the linear wear changes periodically. This is the result of a dynamic process during which the protective layer is formed and periodically removed.

Based on [22,23,24,25,26], the kinetic equation of ZDDP’s decomposition can be expressed as follows:

(6) $$\frac{dD}{dt}=kD^{n}$$

After integration,

(7) $$\left(\frac{1}{t}\left(-n+1\right)\right)D^{-n+1}=k$$

By combining the dependencies in (4), where C = 1/*A_k_k*, and (7), the following is obtained:

(8) $$\left(\left(\frac{1}{t}\left(-n+1\right)\right)\frac{A_{k}\alpha_{iw}}{L_{wo}}P\right)D^{-n+1}+\frac{L_{o}}{L_{wo}}P=W$$

This relationship indicates that the value of linear wear W should depend on the surface concentration of deposits created by the ZDDP additive.

After each test, the wear traces on the surfaces of the discs and balls were analyzed using an EDS elemental analysis. The obtained data are collected in Table 3. For a better understanding of the importance of how the solid surface structure controls the rate of ZDDP decomposition, Table 5 presents the TRB test results for discs covered by a DLC layer.

**Figure 8:**
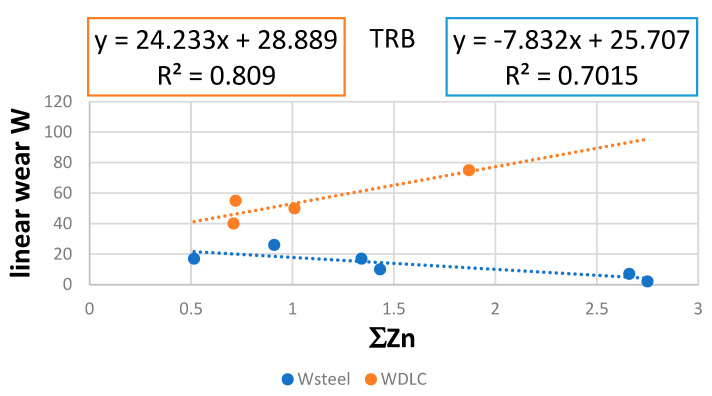
The relationship between linear wear W and Zn concentration in the surface layer of disc and ball together; blue—steel disc, orange—DLC-covered disc.

**Figure 9:**
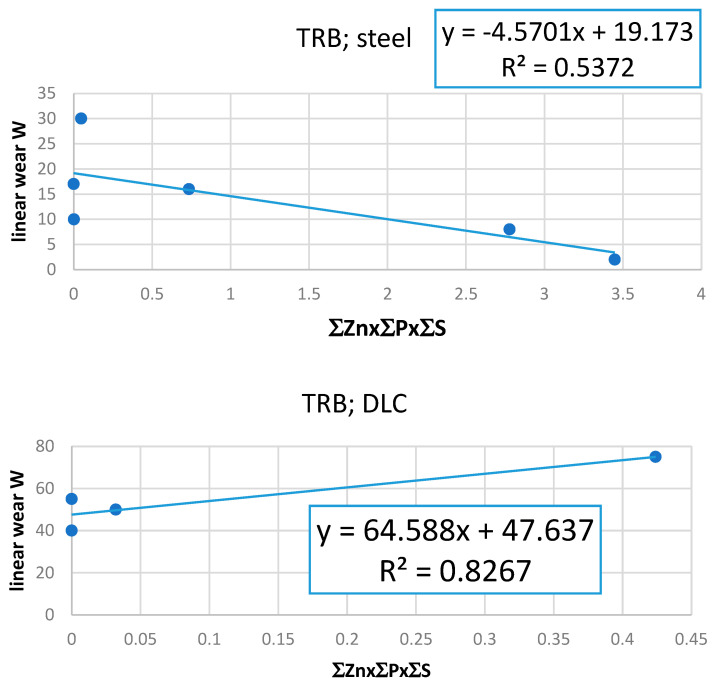
The relationship between linear wear W and deposit concentration in the surface layer of disc and ball together; deposit is expressed as ∑Znx∑Px∑S.

Based on the data presented in Table 5, a linear relationship was found between linear wear W and D, expressed as ∑Zn (Figure 8). It was found that, for steel discs, the more Zn on the discs’ and balls’ surfaces, the lower the value of W. An opposite relationship was found for discs covered by a DLC layer. A similar trend (discs covered by DLC) can be observed when the concentration of deposited D is expressed by ∑*Zn*x∑*P*x∑*S* (Figure 9*)*.

The following dependences were determined experimentally:

- W = −7.832∑Zn + 25.707 for steel disc;
- W = 24.233∑Zn + 28.889 for DLC-covered disc;
- W = −4.5701(∑Znx∑Px∑S) + 19.173 for steel disc;
- W = 64.588(∑Znx∑Px∑S) + 47.637 for DLC-covered disc.

The above empirical dependences indicate that the highest values of the coefficient of determination R² were obtained for linear functions. Consequently, if these functions can be described by Equation (8), the n value should be 0, which means that ZDDP’s reaction rate does not depend on the concentration of ZDDP in the lubricant. What is more, Equation (8) explains why the values of R^2^ are relatively low. There are two reasons:

- The heterogeneity of the surface structure of the wear trace;
- The different values of coefficient of reactivity α_iw_ described for various tested lubricants.

The law values of R^2^ obtained for the relationship between linear wear W and ∑Znx∑Px∑S as well as ∑Px∑S indicate that phosphorus- and sulfur-containing deposits do not influence the linear wear under the conditions of TRB testing.

The results of tribological tests carried out on a TRB tribotester indicate that carbon-based functional nanomaterials control the effectiveness of the ZDDP lubricity additive. CNT makes ZDDP more effective in terms of decreasing linear wear, while graphene, fullerene, and AuCNT make this additive less effective. The influence of CNT, graphene, and fullerene on ZDDP’s effectiveness has been investigated and described in earlier publications. The research described in this paper focused on single-walled nanotubes decorated with Au. The TRB test results indicated the significant role of Au, which completely changes the influence of nanotubes on the effectiveness of ZDDP as a lubricity additive. This can be explained by the participation of Au in electrons’ flow into the carbon skeleton of the nanotube [3,9,10]. In cases where the skeleton is not doped by O or S atoms, Au is preferred to transfer electrons into the carbon skeleton of the nanotube. This process involves intensive energy removal from the environment, in which ZDDP undergoes triboreaction and stores energy inside its structure. Au decoration can make the nanotube structure less effective at energy emission to the reaction space (ZDDP molecules), which decreases the rate of ZDDP’s decomposition.

The postulated earlier mechanism for CNT without decoration having an influence on energy transportation inside the reaction space assumed that CNT takes energy from the solid (steel), stores it, transports it to the space of ZDDP’s triboreaction, and emits it to the molecules of ZDDP.

**Figure 10:**
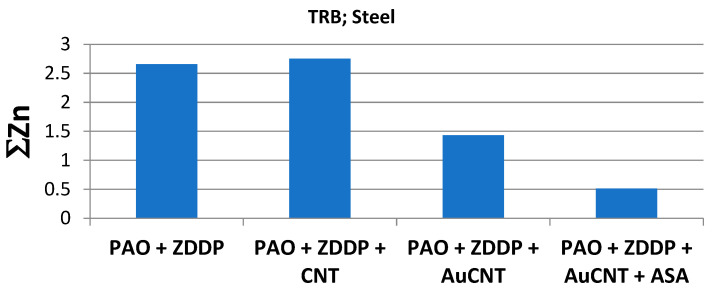
The influence of nanotubes and ASA on the concentration of Zn measured on the surfaces of disc and ball after TRB tests.

This mechanism suggests an increase in the amount of energy provided as support to the molecules of ZDDP during the tribological process and, consequently, an increase in the decomposition rate. It was concluded that, in cases where this mechanism correctly explains the carbon-based nanostructures’ influence on the tribochemical reaction rate, an additive predisposed to conduct electrical charge/energy from the lubricant film to the solid should weaken or completely eliminate the effect of carbon nanomaterials. This was confirmed when the additive ASA was introduced to lubricants containing ZDDP and CNT as well as AuCNT. The data presented above clearly show that, during TRB tests, ASA was responsible for ZDDP’s deactivation and acts as an antiwear additive (linear wear—Figure 3 and Figure 10). As the linear wear increase can be caused by other factors, the concentration of the products of ZDDP’s decomposition (Zn, P, S) directly indicates that ZDDP’s decomposition rate decreases when ASA is added.

### 5.3. The Influence of CNT and ASA on Charge Flow through the Film during TET Tests

The test results presented in Table 3 indicate that ASA does not influence the voltage when it is added to PAO, while it increases the voltage when it is added to the PAO + ZDDP blend. This effect is not as significant as that obtained in the case of the PAO + ZDDP + CNT blend. It was found that the addition of ASA to this blend decreased the voltage, thereby decreasing the influence of CNT on ZDDP’s reactivity.

A similar effect was found when the influence of the pin metal on the voltage was observed in triboelectric tests carried out on a TET stand. CNT being added to the lubricant containing ZDDP changed the influence of the pin metal on the voltage measured during triboelectric tests. The voltage can be treated as the measure of electric charge/energy flow in a tribological system. The exponential functions obtained for PAO and PAO + ZDDP + CNT were significantly different, which indicates quite a different flow of energy through the tribological system. The addition of ASA to the lubricant PAO + ZDDP + CNT changed this function into something similar to that for PAO. This is the same direction of ASA action as was observed during the TRB tests.

**Figure 11:**
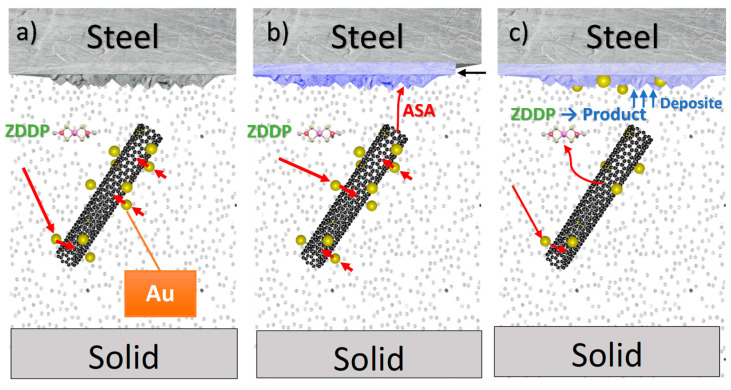
The postulated mechanism for controlling the kinetics of the ZDDP reaction by nanomaterials and ASA. (**a**) Au—Lewis base: activation by internal energy of tribological system returns electrons to CNT, where energy is collected and then (**b**) transferred to the solid element (outside the tribological system) through the structures created by ASA; or (**c**) transferred to ZDDP molecules if there is no ASA in the system.

To summarize, Figure 11 shows the postulated mechanism for controlling the kinetics of the ZDDP reaction by nanomaterials and ASA.

All these conclusions have been based on the results of tribological tests carried out under the same load of 10 N. It was found that linear wear W depends on the applied load but does not change proportionally to it. This suggests that, under different loads, ZDDP formed a protective layer according to various mechanisms. Figure 7 suggests, however, that AuCNT and AuCNT + ASA do not affect the mechanism of protective layer formation, and both obtained curves (ΔW vs. ΔlnP) follow a similar course. Relationship (5) can be helpful in the confirmation of this hypothesis. If ΔW is proportional to ΔlnP, α_i_/C should be constant for different loads (C = A_k_k). ZDDP’s rate constant, k, is the exponential function of 1/T. The load increase should cause a higher temperature, a higher ZDDP rate, and consequently, lower linear wear. Once the load limit is exceeded, the temperature rise effect can no longer compensate for the load and mechanism changes. In relationship (5), the change in the antiwear mechanism is expressed by the change in the α_i_ value. The change in α_i_ vs. ΔlnP is similar for both lubricants tested (containing ZDDP + AuCNT and containing ZDDP + AuCNT + ASA) for both obtained curves (ΔW vs. ΔlnP).

## 6. Conclusions

Based on the results of the tribological research presented in the article and in the previous literature, the following conclusions were formulated:

- Carbon-based nanostructures, i.e., CNTs, AuCNT, graphene, and fullerenes, are able to change the rate of chemical reactions of ZDDP during the tribological process;
- CNTs show the ability to catalyze tribochemical reactions of ZDDP, while graphene and fullerenes are not able to do so effectively;
- AuCNT takes the role of an inhibitor during ZDDP’s triboreaction;
- By discharging electric charges/energy, ASA, in cooperation with CNT and AuCNT, significantly reduces the rate of the ZDDP reaction.

These conclusions are formulated for the tribocatalytic reactions of the ZDDP lubricity additive. The consistency of the conclusions from studies at such diverse sites as TRB and TET with the postulated mechanism of controlling the kinetics of the ZDDP reaction suggests the generalizability of this mechanism to other tribocatalytic reactions. Of course, experimental grounds must be obtained to generalize these conclusions. Developing this concept will allow us to look at tribocatalysis more broadly, examining not only the structure and properties of the catalyst itself but also the reaction environment that is responsible for the transfer of energy to the reactant molecules. It seems that carbon-based nanomaterials may be interesting components of the reaction environment, controlling the efficiency of lubricity.

## Figures and Tables

**Table 1:** Lubricants used in tribological tests.

Base Lubricant	Antiwear/EP Additive; Concentration 1.5 wt.%	Carbon Nanostructures; Concentration 0.005 wt.%	Lubricant Description
PAO 8	-	-	PAO
	ZDDP	-	PAO + ZDDP
	ZDDP	CNT	PAO + ZDDP + CNT
	ZDDP	CNT + ASA	PAO + ZDDP + CNT + ASA
	ZDDP	Graphene	PAO + ZDDP + Graphene
	ZDDP	Fullerene	PAO + ZDDP + Fullerene
	ZDDP	AuCNT	PAO + ZDDP + AuCNT
	ZDDP	AuCNT + ASA	PAO + ZDDP + AuCNT + ASA
	-	AuCNT	PAO + AuCNT

**Table 2:** The values of linear wear after 800 m measured during tribological tests on a TRB tribometer under 10 N load.

Disc Surface	Lubricant	Linear Wear (10 N/800 m) (μm)
TRB *100Cr6*	PAO + ZDDP	8
	PAO + ZDDP + CNT	1
	PAO + ZDDP + Graphene	30
	PAO + ZDDP + Fullerene	17
	PAO + ZDDP + AuCNT	9
	PAO + ZDDP + AuCNT + ASA	17

**Table 3:** Voltage (μV) after 25 s of the TET tribotest.

Lubricant	Voltage (U) (μV)
PEEK—Dural (normal potential −1.1)	
PAO	250
PAO + ZDDP + CNT	633
PAO + ZDDP + CNT+ ASA	1264
PAO + ZDDP	836
PAO + ZDDP + ASA	1174
PAO + ASA	239
PEEK—Al. (normal potential = −1.33)	
PAO	1200
PAO + ZDDP + CNT	652
PAO + ZDDP + CNT+ ASA	1283
PEEK—Cu (normal potential = +0.34)	
PAO	4
PAO + ZDDP + CNT	250
PAO + ZDDP + CNT+ ASA	50

**Table 4:** The relationships between the values of the voltage TET U and the normal potential of the pin material E^o^_Me_.

Lubricant	Experimentally Obtained Exponential Function
PAO	$U=11.3e^{-3.23{E^{o}}_{Me}}$
PAO + ZDDP + CNT	$U=309.0e^{-0.60{E^{o}}_{Me}}$
PAO + ZDDP + CNT + ASA	$U=103.8e^{-2.05{E^{o}}_{Me}}$

**Table 5:** The values of linear wear measured during tribological tests on TRB tribometer and Zn concentration on the surface of the ball and disc together (∑Zn) [18,19], as well as (∑Znx∑Px∑ S)/(∑Px∑S).

Disc Surface	Lubricant	Linear Wear (μm)	*∑Zn* (wt.%)	(∑*Zn*x∑*P*x∑*S*) (wt.%)/(∑*P*x∑*S*) (wt.%)
TRB *100Cr6*	PAO + ZDDP	7	2.66	2.778/1.46
	PAO + ZDDP + CNT	2	2.75	3.673/1.33
	PAO + ZDDP + Graphene	29	0.91	0.054/0.06
	PAO + ZDDP + Fullerene	17	1.34	0.725/0.54
	PAO + ZDDP + AuCNT	10	1.432	0.123/0.086
	PAO + ZDDP + AuCNT + ASA	17	0.515	0.107/0.207
TRB DLC	PAO + ZDDP	55	0.72	0
	PAO + ZDDP + CNT	75	1.87	0.424
	PAO + ZDDP + Graphene	50	1.01	0.032
	PAO + ZDDP + Fullerene	40	0.71	0

## Data Availability

Data are contained within the article.

## Nomenclature

*A*	coefficient (constant) for given mechanism of reactions
α_i_	reactivity coefficient of tested reactants
*E^o^_Me_*	normal potential of pin material (TET stand) (V)
*K*	rate constant of reaction, which stimulates the observed process
*L*	energy supplied from environment to the system (J)
*L*_0_	energy supplied from environment to the system—reference value (J)
*P*	applied load
*R*	gas constant
*R* ^2^	coefficient of determination
*t*	duration of combustion process (s)
*T*	average temperature of reaction system (K).

## Acronyms

ASA	antistatic additive for jet fuels
AuCNT	carbon nanotubes decorated by Au
C60	fullerene
CNTs	carbon nanotubes
PAO	polyalphaolefins
TET	triboelectric tribometer
TRB	Anton Paar tribometer
ZDDP	zinc dialkyldithiophosphate
